# Incidence of retinopathy of prematurity type 1 and type 2 in a regional Hospital of Social Security in the state of Queretaro, Mexico (2017–2018)

**DOI:** 10.1186/s12886-019-1095-0

**Published:** 2019-04-15

**Authors:** Roger Acevedo-Castellón, Paulina Ramírez-Neria, Renata García-Franco

**Affiliations:** Instituto Mexicano de Oftalmología IAP, Circuito de Estadio de Corregidora, sin número, Santiago de Querétaro, Querétaro, Mexico

**Keywords:** Retinopathy of prematurity, Type 1 ROP, Type 2 ROP

## Abstract

**Background:**

Retinopathy of prematurity (ROP), the primary cause of blindness in children, is a potential complication for 7.7% of live births in Mexico. Given that less than one-third of all neonatal intensive care units follow Mexican National ROP guidelines, there have been few reports regarding the incidences of types 1 and 2 ROP.

**Methods:**

This was a retrospective study that investigated the incidence and onset of ROP in a representative sample of children in Mexico. We analyzed the results obtained by the ROP Detection and Treatment Program, compliant with the Mexican National ROP guidelines, over a 1-year period. This study included 132 children who were born prematurely, were initially screened between October 2, 2017 and October 1, 2018, and underwent follow-up based on their risk group (in accordance with the Mexican National ROP guidelines).

**Results:**

The mean gestational age (GA) at birth was 32 weeks and 3 days (32w3d) (95% CI, ± 3 days), and the mean birth weight (BW) was 1594 g (95% CI, ± 96 g). The clinical features were as follows: 36.4% had immature retina without ROP, 22.0% had mild ROP, 5.3% had type 2 ROP, 27.3% had type 1 ROP, and 1.5% had advanced disease. Premature children with ROP requiring treatment (i.e., type 1 ROP + advanced ROP) were born at an MGA of 30w4d (95% CI, ± 5d; range, 26–35 weeks); their MBW was 1316 g (95% CI, ± 110 g; range, 830–2220 g). Diagnosis of ROP requiring treatment was made at a mean postmenstrual age (PMA) of 37w3d (95% CI, ± 5d; range, 31w1d to 42w1d).

**Conclusion:**

In Mexico, screening and close ophthalmological follow-up of children who present with risk factors of birth weight < 1750 g and gestational age ≤ 34 weeks, both of which are observed more frequently in children with type 1 ROP, appears essential for implementing timely treatments (within 72 h). This is particularly important for children with PMA between 36 and 38 weeks, which is considered to be the peak age for disease stages that require timely intervention.

## Background

Retinopathy of prematurity (ROP), a pathological process that affects immature retinal tissue, can progress to tractional retinal detachment, which results in visual impairment [1]. The available evidence suggests that there is considerable variation in the population of premature babies at risk of ROP; larger, more mature babies develop treatable disease more frequently in low and middle income countries than in industrialized countries [2].

In Mexico, 7.7% of live births attended by the Mexican Social Security Institute (IMSS, the main medical service provider) are premature [3]. Thus, it is important to improve the conditions of care in neonatal intensive care units (NICUs), as well as the detection of ROP and subsequent implementation of timely treatment, if needed [4]. In Mexico, the rate of infant mortality in children under 1 year of age was 11.5 per 1000 live births in 2017 [5]; thus, the risk of blindness due to ROP is very high due to adverse neonatal outcomes [6]. Recently, in the Early Treatment of Retinopathy of Prematurity Randomized Trial, researchers confirmed the efficacy of treatment for high-risk pre-threshold ROP (recategorized as type 1 ROP); moreover, they redefined the indications for treatment and established clinical classifications of types 1 and 2 ROP [1]. Following this update, it is important to identify the incidences of these conditions in health centers where ROP screening activities are performed.

Evaluation of the population at risk of ROP should be performed on the basis of gestational age (GA) at birth, as well as presence and severity of systemic disease. In Mexico, compulsory screening for ROP has been established for children born at or prior to 34 weeks GA, as well as for those with a birth weight (BW) of < 1750 g [4]. In general terms, the first evaluation should be performed in children at 31 weeks postmenstrual age (PMA; GA at birth + chronological age of the child after birth) if their GA at birth is ≤27 weeks. Otherwise, the first evaluation should be performed 4 weeks after birth. Subsequent assessments should be scheduled with intervals ranging from less than 1 week to 3 weeks, depending on the degree of severity found in previous assessments. Furthermore, follow-up can be discontinued at 35 weeks PMA if vascularization has reached Zone III in eyes that have not previously shown any indication of ROP. If normal vascularization is not observed, surveillance should be extended up to 45 weeks PMA, or up to 65 weeks PMA in patients who have been treated with anti-angiogenic drugs because of the risk of late recurrence [1].

In Mexico, less than one-third of the NICUs have a program that complies with the Mexican National ROP guidelines; therefore, there have been few reports regarding the incidence of types 1 and 2 ROP [7]. For the year 2017, the National Institute of Statistics and Geography of Mexico reported 2,234,039 births throughout the nation [5]. Of these, 4398 were registered in Querétaro, and 97.7% were attended in a clinic or hospital by a physician or obstetric nurse. The IMSS hosted 9954 live births in its health centers (in the State of Querétaro, 23.7% of births took place in a hospital or clinic), and 733 children were classified as premature (7.6%) [8]. The Mexican Institute of Ophthalmology IAP (IMO) launched its Screening, Detection, and Treatment Program for ROP in Querétaro in 2017. Since that time, most children are initially screened in the NICU of the Regional Hospital Number 1 of the IMSS in Querétaro. This descriptive study aimed to determine the incidences of types 1 and 2 ROP among premature Mexican children born in Querétaro, and therefore reports the results obtained by screening children recruited in this center.

## Methods

This was a retrospective study of the results of the ROP Screening, Detection, and Treatment Program, directed by the IMO and the IMSS in the NICU located within the facilities of the Regional Hospital No. 1 of Querétaro. This NICU is a level III unit that welcomes students from local universities for all health disciplines (medicine, nursing, microbiology, and pharmacy), and has its own ophthalmology department. It is organized into three modules: critical care, intermediate care, and weight gain. The data analyzed in this study were obtained from initial ROP screenings that were performed between October 2, 2017 and October 1, 2018.

Children were screened if their BW was < 1750 g or if they were born at ≤34 weeks GA; children were also screened if they showed other risk factors, as identified by their pediatrician [4]. Pupillary dilation was induced using tropicamide (0.8%)–phenylephrine (5%). Thereafter, retinal screening examinations were performed by using binocular indirect ophthalmoscopy with a 28-diopter lens, Alfonso Eye lid speculum, and Flynn scleral depressor (as needed). The screening was performed by an ophthalmologist who had experience in accurate identification of the location and characteristic retinal changes of ROP [1].

All children with type 1 ROP were primarily treated with intravitreal ranibizumab (0.25 mg / 0.025 mL) within 24 h after diagnosis; those who exhibited advanced ROP underwent vitrectomy within 1 week after diagnosis. Informed consent was obtained from the parents of all patients. Follow-up examinations were recommended by the examining ophthalmologist on the basis of retinal findings, which were classified in accordance with guidelines of the “International Classification of Retinopathy of Prematurity Revisited” [1].

Data of children for whom clinical follow-up was not completed in accordance with clinical practice guidelines were analyzed separately, because their actual clinical evolution was unknown; these children failed to attend their screening appointments following discharge from the NICU. Data of children for whom appropriate clinical follow-up was completed were categorized as follows: 1) children who were followed up until 35 weeks PMA, and who presented with immature retina in zone III and no previous history of changes due to retinopathy (stages different than zero or plus disease) in zones I and II; 2) children who were followed up until 45 weeks PMA, and who had not reached retinal maturity, did not show retinal changes due to ROP in any of their evaluations, and did not require treatment (mild ROP and type 2 ROP); and 3) children who were followed up until 65 weeks PMA, and who developed type 1 ROP, received intravitreal ranibizumab, and showed regression [1]. Infants who underwent vitrectomy due to advanced ROP then completed individualized follow-up, based on their evolution of disease after surgery.

The registry of the evaluations used to extract the data was kept both in physical format in a logbook and in electronic format in a Microsoft Excel® file (Microsoft, Redmond, WA, USA). The eyes of the children were classified as follows:

- **mature retina:** complete retinal vascularization in the initial evaluation (retinal blood vessels that reached the periphery in the proximity of the ora serrata, as observed in healthy adults).
- **immature retina:** incomplete retinal vascularization without evidence of ROP in any evaluations.
- **mild ROP:** ROP changes that do not meet the criteria of types 1 or 2 ROP, or of advanced stages (i.e., stages 4 or 5).
- **type 2 ROP:** stage 1 or 2 ROP in zone I without “plus disease,” or stage 3 ROP in zone II without plus disease.
- **type 1 ROP:** stage 1 or 2 ROP in zone I with plus disease, stage 3 ROP in zone I with or without plus disease, or stage 2 ROP in zone II with plus disease.
- **Advanced ROP:** stages 4A, 4B, 5A, or 5B ROP.

Descriptive statistical analyses with measures for central tendency and dispersion (mean, confidence intervals, and ranges), and inferential statistical analysis with one-way ANOVA test (alpha = 0.05) were performed in Microsoft Excel®; differences were assessed in relation to mean gestational age (MGA), mean birth weight (MBW), mean postmenstrual age (MPMA) and total number of evaluations performed to complete follow up between the two groups: premature children who did not require treatment (non-RT: mature retina, immature retina, mild ROP, and type 2 ROP) and those who required treatment (RT: type 1 ROP, and advanced ROP). Means ± Standard Deviation (SD) are presented in statistical analysis with one-way ANOVA within the text, and Means ± CI 95% within the results section in Table 1 to facilitate comparison between groups.

**Table 1:** Birth weight, gestational age and postmentrual age at the time of diagnosis or presentation according to categories of severity of ROP

	*Prematures*		BW (g) (CI 95%) *[range]*	GA (CI 95%) *[range]*	1st Ev^2^ (CI 95%) *[range]*	PMA (CI 95%) *[range]*	Last Ev^2^ (CI 95%) *[range]*	No Ev^2^ (CI 95%) *[range]*
	*n*	*%*						
*non RT* ^1^	94	71.2%	1707 ± 121	33w 2d ± 0w 4d	37w 3d ± 0w 5d	38w 4d ± 0w 5d	43w 6d ± 1w 2d	3.62 ± 0.65
			*[630, 4700]*	*[27w 0d, 42w 0d]*	*[31w 0d, 51w 0d]*	*[33w 0d, 50w 6d]*	*[35w 0d, 71w 0d]*	*[1, 15]*
*Mature Retina*	10	7.6%	2331 ± 553	36w 2d ± 1w 2d	40w 5d ± 1w 6d	40w 5d ± 1w 6d	40w 5d ± 1w 6d	1.00 ± 0
			*[1720, 4700]*	*[32w 0d, 40w 0d]*	*[34w 6d, 44w 2d]*	*[34w 6d, 44w 2d]*	*[34w 6d, 44w 2d]*	[1]
*Immature Retina*	48	36.4%	1801 ± 151	33w 5d ± 0w 5d	38w 2d ± 1w 0d	38w 2d ± 1w 0d	41w 6d ± 1w 3d	2.23 ± 0.48
			*[1010, 3600]*	*[28w 0d, 42w 0d]*	*[32w 0d, 51w 0d]*	*[33w 0d, 50w 6d]*	*[35w 0d, 56w 0d]*	[1, 9]
*Mild ROP*	29	22.0%	1433 ± 141	31w 6d ± 0w 6d	35w 5d ± 1w 2d	38w 4d ± 1w 2d	47w 6d ± 2w 4d	6.31 ± 1.27
			*[630, 2560]*	*[27w 0d, 36w 0d]*	*[31w 0d, 50w 0d]*	*[33w 4d, 49w 5d]*	*[38w 0d, 71w 0d]*	*[1, 15]*
*Type 2 ROP*	7	5.3%	1304 ± 278	31w 0d ± 0w 5d	34w 5d ± 1w 3d	37w 0d ± 3w 0d	45w 3d ± 6w 1d	5.71 ± 2.52
			*[950, 1830]*	*[29w 0d, 32w 0d]*	*[31w 0d, 37w 0d]*	*[33w 5d, 45w 4d]*	*[35w 0d, 55w 0d]*	[2, 12]
*RT* ^1^	38	28.8%	1316 ± 110	30w 4d ± 0w 5d	35w 1d ± 0w 6d	37w 3d ± 0w 5d	47w 3d ± 2w 3d	8.84 ± 1.13
			*[830, 2220]*	*[26w 0d, 35w 0d]*	*[29w 0d, 40w 0d]*	*[31w 1d, 42w 1d]*	*[33w 0d, 66w 0d]*	*[3, 16]*
*Type 1 ROP*	36	27.3%	1310 ± 112	30w 4d ± 0w 5d	34w 6d ± 0w 5d	37w 2d ± 0w 5d	46w 4d ± 2w 1d	8.91 ± 1.13
			*[830, 2220]*	*[26w 0d, 35w 0d]*	*[29w 0d, 40w 0d]*	*[31w 1d, 42w 1d]*	*[33w 0d, 66w 0d]*	*[3, 16]*
*Advanced ROP*	2	1.5%	1425 ± 735	29w 3d ± 4w 6 d	39w 1d ± 1w 6d	39w 1d ± 1w 6d	63w 0d ± 5w 6d	5.00 ± 1.96
			*[1050, 1800]*	*[27w 0d, 32w 0d]*	*[38w 0d, 40w 0d]*	*[38w 2d, 40w 1d]*	*[60w 0d, 66w 0d]*	[4, 6]

^1^ *RT: requiring treatment*

^2^ *Ev: evaluation*

To avoid potential registration errors that could affect the calculations, consistency between physical and electronic records was meticulously checked.

Evaluations performed in this retrospective study were regarded as epidemiological surveillance and were therefore mandatory nationwide, in accordance with the official Mexican ROP Guidelines (NOM-034-SSA2–2013) issued by the Ministry of Health [9]. However, we ensured that informed consent was obtained from parents to screen their children, in order to publish these data. In addition, this research protocol adhered to the guidelines of the Declaration of Helsinki, and was approved by the Institutional Scientific Ethics Committee of the IMO on July 27, 2018.

## Results

**Fig. 1:**
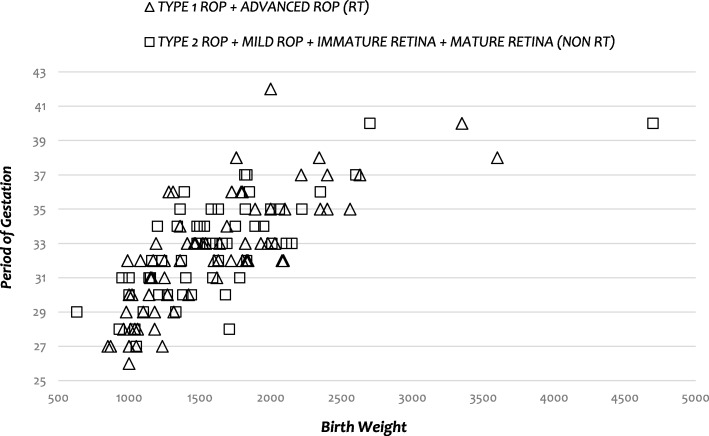
Gestational age by birth weight distribution of premature children who completed the surveillance period for retinopathy of prematurity, in accordance with treatment requirements

The analyses included 132 of 159 premature children in whom clinical follow-up was completed. Their MGA at birth was 32 weeks and 3 days (32w3d) (95% confidence interval [CI], ± 3 days; range, 26–42 weeks). Their MBW was 1594 g (95% CI, ± 96 g; range, 630–4700 g). Children who completed follow-up underwent a mean of 5.1 (95% CI, ± 0.7) assessments, whereas those who did not complete follow-up underwent only 2.0 (95% CI, ± 0.5) assessments. The findings regarding infants who underwent complete follow-up were as follows: 7.6% showed complete retinal vascularization (i.e., mature retinal vasculature) during the initial evaluation; 36.4% had immature vessels and did not develop ROP; 22.0% had mild ROP (i.e., they did not develop types 1 or 2 ROP); 5.3% had type 2 ROP; 27.3% had type 1 ROP, and 1.5% had advanced ROP before any treatment was administered (Table 1). Compared to RT infants, non-RT infants were heavier (MBW 1707 [SD, ± 597] versus 1316 [SD, ± 346], *p* < 0.0001), older at birth (MGA 33w 2d [SD, ± 3w] versus 30w 4d [SD, ± 2w 3d], *p* = 0.0002), and needed fewer evaluations to complete the surveillance period (3.62 [SD, ± 3.20] versus 8.84 [SD, ± 3.47], *p* < 0.0001). There was no statistically significant differences regarding mean PMA between these two groups (*p* = 0.08) (Table 1)(Fig. 1).

Considering the degree of development of the retinal vasculature found in the initial evaluation, premature infants with immature retina frequently demonstrated vascularization that reached zone III (64.6%), while infants with mild ROP frequently demonstrated vessels that extended to zone II (75.9%). For infants with type 2 ROP, an even distribution between zones I and II was observed at the time of diagnosis; for infants with type 1 ROP, retinal vascularization had reached zone II in most cases at the time of diagnosis (94.4%) (Table 2). For infants with mild ROP, the most frequent finding was stage 1 ROP (58.6%); for infants with types 1 and 2 ROP, there was an even distribution between stages 2 and 3 ROP at the time of diagnosis. Two infants with advanced ROP showed stage 4 disease (Table 3).

**Table 2:** Identified zone at the time of diagnosis according to categories of severity of ROP

	*ZONE*						
	*I*		*II*		*III*		*TOTAL*
	*n*	*%*	*n*	*%*	*n*	*%*	*n*
*non RT* ^1^	5	6.0%	41	48.8%	38	45.2%	84
*Immature Retina*	1	*2.1%*	16	*33.3%*	31	*64.6%*	*48*
*Mild ROP*	0	*0.0%*	22	*75.9%*	7	*24.1%*	29
*Type 2 ROP*	4	*57.1%*	3	*42.9%*	–	*–*	*7*
*RT* ^1^	3	7.9%	35	92.1%	0	0.0%	38
*Type 1 ROP*	2	*5.6%*	34	*94.4%*	–	*–*	*36*
*Advanced ROP*	1	*50.0%*	1	*50.0%*	–	*–*	*2*
*All*	8	*6.6%*	76	*62.3%*	38	*31.1%*	122

^1^ *RT: requiring treatment*

**Table 3:** Identified ROP stage at the time of diagnosis, according to categories of severity of ROP

	*STAGE*										
	*1*		*2*		*3*		*4A*		*4B*		*TOTAL*
	*n*	*%*	*n*	*%*	*n*	*%*	*n*	*%*	*n*	*%*	*n*
*non RT* ^1^	18	50.0%	14	38.9%	4	11.1%	–	–	–	–	36
*Mild ROP*	17	*58.6%*	11	*37.9%*	1	*3.4%*	–	–	–	–	*29*
*Type 2 ROP*	1	*14.3%*	3	*42.9%*	3	*42.9%*	–	–	–	–	*7*
*RT* ^1^	1	2.6%	17	44.7%	18	47.4%	1	2.6%	1	2.6%	38
*Type 1 ROP*	1	*2.8%*	17	*47.2%*	18	*50.0%*	–	–	–	–	*36*
*Advanced ROP*	–	–	–	–	–	–	1	*50.0%*	1	*50.0%*	*2*
*All*	19	25.7%	31	41.9%	22	29.7%	1	1.4%	1	1.4%	*74*

^1^ *RT: requiring treatment*

Aggressive posterior ROP was identified in three premature children, who had an MGA of 27w4d (95% CI, ± 4d) and an MBW of 1075 (95% CI, ± 173 g). Two of these three had stage 3+ disease in posterior zone II, and one had stage 3+ disease in zone I. They were diagnose with aggressive posterior ROP at a mean PMA of 35w1d (95% CI, ± 1w 6d; range, 33w3d to 36w4d); as they were within the type 1 ROP group, they received intravitreal ranibizumab as primary treatment. The initial indications for ROP screening in 86.8% of premature children who finally required and received treatment (type 1 ROP and advanced ROP) were low BW and low GA. Only 2.6% of the infants who developed ROP that required treatment had BW of > 1750 g and GA > 34 weeks. These infants were screened for ROP on the basis of the pediatrician’s request, because of the presence of other risk factors (i.e., sepsis and oxygen exposure) (Table 4).

**Table 4:** Screening criteria used in premature children for recruitment according to categories of severity of ROP

	*SCREENING CRITERIA*								
	*BW < 1750 g only*		*GA ≤ 34w only*		*BW < 1750 g and GA ≤ 34w*		*Other*		*TOTAL*
	*n*	*%*	*n*	*%*	*n*	*%*	*n*	*%*	*n*
*non RT* ^1^	7	7.4%	13	13.8%	48	51.1%	26	27.7%	94
*Mature Retina*	1	*10.0%*	1	*10.0%*	1	*10.0%*	7	*70.0%*	*10*
*Immature Retina*	4	*8.3%*	8	*16.7%*	20	*41.7%*	16	*33.3%*	*48*
*Mild ROP*	2	*6.9%*	2	*6.9%*	22	*75.9%*	3	*10.3%*	*29*
*Type 2 ROP*	0	*0.0%*	2	*28.6%*	5	*71.4%*	0	*0.0%*	*7*
*RT* ^1^	0	0.0%	4	10.5%	33	86.8%	1	2.6%	38
*Type 1 ROP*	0	*0.0%*	3	*8.3%*	32	*88.9%*	1	*2.8%*	*36*
*Advanced ROP*	0	*0.0%*	1	*50.0%*	1	*50.0%*	0	*0.0%*	*2*
*All*	7	5.3%	17	12.9%	81	61.4%	27	20.5%	*132*

^1^ *RT: requiring treatment*

Regarding premature children who did not complete the surveillance period (17%), at their last visit at the NICU they showed the following pathophysiologies: mild ROP (66.7%) or immature retina (33.3%) (Table 5). The initial indications for screening in these children were BW < 1750 g or GA ≤ 34 weeks (98.1%).

**Table 5:** Birth weight, gestational age and postmentrual age at the time of diagnosis or presentation, according to categories of severity of ROP within premature children who did not complete surveillance period

	Prematures		BW (g) (CI 95%)	GA (CI 95%)	Last Evaluation (CI 95%)
	n	%			
Immature Retina	9	33.3%	1761 ± 327	32w 6d ± 1w 3d	37w 3d ± 1w 0d
Mild ROP	18	66.7%	1615 ± 127	31w 5d ± 0w 6d	37w 2d ± 0w 6d

## Discussion

The NICU of the Regional Hospital Number 1 of the State of Querétaro, attended by the IMSS, reported 733 live premature births (GA < 37 weeks) in 2017. In this institution, a total of 159 children with an MGA of 32w3d ± 3d were screened for ROP during that year, on the basis of the presence of risk factors for the disease. Only 132 children (83%) completed the surveillance period. Although the hospital staff arranged and scheduled the first post-discharge outpatient ophthalmology examination [1], a subset of patients were lost to follow-up. Future studies should address the factors involved in this lack of follow-up.

The most recent statistical values indicate that approximately 21.7% of the children born prematurely in the State of Querétaro and attended by the IMSS are also admitted to the NICU of Regional Hospital Number 1 and screened for ROP on the basis of the presence of risk factors for the disease. In total, 27.3% of the children who were screened demonstrated type 1 ROP; this incidence is lower than that reported by Zepeda et al. (44%) [10]. This difference may be explained by inter-regional differences in the quality of neonatal care, even within the same social security system [7]. Moreover, evolution of healthcare programs in the past 7 years, which has improved care conditions in NICUs, may also have contributed to this reduced incidence [6]. Indeed, the infant mortality rate in Mexico has decreased by one-half unit per year since 2010 (14.9/1000 live births), reaching 11.5/1000 live births in 2017 [5].

Type 1 ROP onset began at approximately 37w2d (95% CI, ± 5d) PMA, which represents a difference of 1 week relative to that reported by Quinn et al. (36w3d) [11]. This difference might have arisen because the data analyzed in this study (involving 29 health centers in the USA and Canada) were obtained from a more immature population of premature children, compared to that in our study, with an MBW of 1100 g and standard deviation (SD) of ±363 g, and an MGA of 28w and SD of ±3w [11]. The characteristics of children with type 1 ROP in our study included zone II with plus disease (94.4%); these differed from the characteristics reported by Quinn et al., who indicated a frequency of type 1 ROP of 77.3% [11]. This difference could be due to a more frequent presentation of type 1 ROP cases with zone I disease (22.7%) [11] in the US and Canada, compared with that in our study (5.6%), which may be related to the higher degree of immaturity of premature infants analyzed (as mentioned above) resulting from improved survival of premature babies in these countries. Indeed, improved quality of care in NICUs in the USA and Canada is reflected by their lower infant mortality rates (5.8/1000 live births and 4.5/1000 live births, respectively), compared to those in Mexico in 2017 (11.6/1000 live births) [12]. Posterior aggressive ROP was found in 2.3% of premature children screened; this incidence was higher than that reported by Quinn et al. (0.2%) [11], which may be a result of similar factors as those presented above for type 1 ROP.

Type 2 ROP was observed in 4.5% of children who underwent screening, which is similar to the findings of Zepeda et al. (4.3%) [10], but differs from the findings of Quinn et al. (6.3%) [11]. This could be a result of less frequent evolution of type 2 ROP to type 1 ROP in the USA and Canada, due to improved control of risk factors (e.g., liberal oxygen exposure without the use of blenders in the NICUs) [13]. Mild ROP was found in 21.6% of children who underwent screening, which was similar to the incidence reported by Zepeda et al. in a NICU in Guadalajara, México between 2005 and 2010 (24.4%) [10], as well as the incidence reported by Quinn et al. (30.6%) [11].

The cumulative proportion of categories in which ROP was not found (i.e., mature retina and immature retina, 43.9%), was greater than that of each of the other categories in which ROP was found, similar to the findings by Quinn et al. in the Secondary Analysis of the Postnatal Growth and Retinopathy of Prematurity Study [11]—that study described the largest cohort of infants who underwent ROP screening thus far; the predominant category was that of no ROP in either eye (56.9%).

The strength of the present study was its complete record of program data with assessments made in both physical and digital formats. The primary limitation, due to its retrospective nature, was the lack of standardized training among ophthalmologists who performed the screening, which may have led to considerable interobserver variability, and may represent an important source of bias during classification.

## Conclusions

The high incidence of type 1 ROP estimated in this study is a clear indicator of the need to improve the conditions of care in the NICU, such as the administration of supplemental oxygen with the aid of blenders. Screening and close ophthalmological follow-up of children who present with combined risk factors of low BW (< 1750 g) and GA of < 34 weeks, observed more frequently in children who developed type 1 ROP, is essential for the provision of timely treatment. Follow-up should be especially frequent between 36 and 38 weeks PMA, representing the peak age for disease occurrence based on the data obtained in this study.

## Abbreviations

BW: Birth weight
GA: Gestational age
MBW: Mean birth weight
MGA: Mean gestational age
NICU: Neonatal intensive care unit
PMA: Post menstrual age
ROP: Retinopathy of prematurity
RT: Requiring treatment
